# Survivors of avalanche accidents: posttraumatic stress disorder symptoms and quality of life: a multicentre study

**DOI:** 10.1186/s13049-021-00912-3

**Published:** 2021-07-19

**Authors:** Charlotte Léonard, Anaëlle Charriau-Perret, Guillaume Debaty, Loïc Belle, Cécile Ricard, Caroline Sanchez, Pierre-Marie Dupré, Gregory Panoff, Thierry Bougerol, Damien Viglino, Marc Blancher, the Northern French-Alps Emergency Network (“RENAU”)

**Affiliations:** 1 Grenoble University Hospital, Emergency Department and Mobile Intensive Care Unit, University Grenoble Alpes, SAMU 38, 38 000 Grenoble, France; 2 TIMC-IMAG laboratory Team PRETA, CNRS UMR 5525, University Grenoble Alpes, Grenoble, France; 3 Cardiac Intensive Care Unit, Annecy-Genevois Hospital, Annecy, France; 4 North Alpine Emergency Network Department (RENAU), Annecy, France; 5 Peloton de Gendarmerie de Haute Montagne (PGHM Mountain Rescue), Chamonix-Mont-Blanc, France; 6 Compagnie Républicaine de Sécurité (CRS-Alpes Montain Rescue), Les Bossons, Chamonix, France; 7 Institute of Neurosciences, Inserm U836, Grenoble Alpes University, Grenoble, France; 8 Hypoxia-Physiopathology Laboratory HP2, INSERM U1300, Grenoble Alpes University, Grenoble, France

**Keywords:** Post-traumatic stress disorder, Avalanche, Quality of life, SF-12, And impact of event scale revised (IES-R)

## Abstract

**Background:**

As any traumatic event, avalanches could trigger psychological disorders on survivors. Our objectives were to determine the prevalence of post-traumatic stress disorder among avalanche survivors and to evaluate post-traumatic stress disorder risks factors as well as the impact on quality of life.

**Methods:**

A multicentre study was conducted in victims included in the North Alpine Avalanche Registry from 2014 to 2018. Data were collected through a standard questionnaire during semi-directed phone interviews. The primary outcome was the total score on the Impact of Event Scale Revised. Secondary outcomes were the Mental Component Scale and the Physical Component Scale scores of the Short Form 12 questionnaire.

**Results:**

During the study period, 132 of 211 victims survived. Among the 107 victims included, 55 (51.4%) phone interviews were obtained. Six patients (10.9, 95% CI 1.76–20.05) had an Impact of Event Scale Revised score ≥ 33 indicating a strong probability for post-traumatic stress disorder. Median Mental Component Scale score was 39.0 (IQR 30.5–46.3) for post-traumatic stress disorder patients and 40.1 (IQR 36.5–43.4) for non post-traumatic stress disorder (*p* = 0.76). Median Physical Component Scale score was 39.4 (37.2–44.3) for post-traumatic stress disorder patients and 44.2 (39.1–46.8) for non post-traumatic stress disorder (*p* = 0.39). No significant difference in the quality of life in both populations was observed, and no independent risk factors of post-traumatic stress disorder was identified.

**Conclusion:**

Avalanche accidents may induce post-traumatic stress disorders among survivors in a comparable prevalence to the most traumatic event already studied. Early recognition and preventive measures should be set up in order to reduce the psychological burden in these victims.

**Trial registration:**

NCT03936738.

## Introduction

Post-traumatic stress disorder (PTSD) is a relatively recent pathological entity, emerging from war trauma victims and recently revised in the 5th Edition of the Diagnostic and Statistical Manual of mental disorders (DSM-V). It can affect a person who has faced death, a death threat, or a serious injury. There are four specific symptoms of this disorder: intrusion (or reliving the trauma), avoidance, significant changes in mood and cognitive abilities, and hyper arousal. The diagnosis of PTSD is confirmed if symptoms are present more than one month after the traumatic event [1]. About 9.2% of individuals exposed to a traumatic experience will develop post-traumatic stress, but prevalence varies depending on the type and the severity of trauma [2, 3]. PTSD can occur months or even years after the trauma [4]. Without diagnosis and treatment, PTSD can lead to functional impairment or disability. It can affect all domains of health statute: social, professional and somatic. As a result, quality of life can be deeply affected [5–7]. Early recognition of PTSD could prevent disorders and its consequences for some victims [8].

Several studies have identified the occurrence of post-traumatic stress following traumatic events such as road accidents, natural disasters or among war veterans [5, 9, 10]. Very few studies focused on the occurrence of post-traumatic stress in avalanche victims [11–16]. Due to its brutal, unexpected and often fatal characteristics, an avalanche is likely to cause intense stress for a survivor. In a prospective study, the prevalence of post-traumatic stress disorder in soldiers affected by a fatal avalanche was 12% four months after the episode [13]. After a major avalanche in 1995 that buried an entire Icelandic village, 40% of survivors suffered from PTSD at fourteen months and 25% of survivors presented significant psychological distress [11]. Sixteen years after an avalanche, a study found 16% of PTSD among survivors, illustrating the long-term persistence of these symptoms [14]. These studies focused on avalanche victims after a natural disaster event. To date, no data is available on the prevalence of post-traumatic stress disorder and its consequences among avalanche victims in recreational sports. Each year in the French Northern-Alps region, between 40 and 80 persons are receiving medical care after being involved in an avalanche incident. Since 2014, prospective data on pre-hospital and intra-hospital management of avalanche victims are collected in the Northern French-Alps Avalanche Registry (RENAAV). The knowledge about the psychological impact of an avalanche on survivors remaining weak, the aim of this study was to assess the prevalence of PTSD symptoms in avalanche victims included in the RENAAV. We also evaluated the impact on quality of life, explored potential risk factors for PTSD in this population, and finally researched consequences on further sports practice by victims.

## Methods

### Study design and setting

All persons involved in an avalanche and from whom medical (or paramedic) examination is available are prospectively included in the North Alpine Avalanche Registry (RENAAV). The RENAAV is a prospective multicentre registry. It collects data about all avalanche victims managed by medical mountain rescue teams in Northern French-Alps Emergency Network (RENAU). Nine hospitals of the Rhône-Alps region participated: Annecy Hospital, Albertville Hospital, Briançon Hospital, Bourg-Saint-Maurice Hospital, Chambéry Hospital, Grenoble University Hospital, Sallanches Hospital, Saint-Jean-de-Maurienne Hospital, and Gap Hospital. This registry contributes to improve the quality of advanced medical care for the victims. A person was considered to be involved in an avalanche when he or she was in direct contact with the avalanche or its snow spray. All French speaking adults included in the RENAAV between 01 December 2014 and 31 May 2018 were eligible to the study.

### Measurements

Out-of-hospital and intra-hospital management data were extracted from the North Alpine Avalanche Registry (RENAAV) and medical charts. Post-traumatic stress symptoms and quality of life were assessed during phone interviews in 2019. Eligible patients were contacted in random order to present study goals and to obtain their oral consent. After three contact failures, people were considered as non-responders. The main interview was planned separately if consent was obtained.

The primary end point was the Impact of Event Scale Revised (IES-R) total score. The IES-R is a self-reported measure assessing the subjective distress caused by a traumatic event. It contains the 22 original IES items and seven additional items related to the hyperarousal symptoms of the PTSD. A 5-points Likert scale is filled for each item in relation to their experiences during the preceding 7 days. The French version of the IES-R is available since 1998, with validated psychometrics properties [17–19]. Subjects were divided into two groups: “PTSD +” with a very high probability of PTSD corresponding to subjects with a total IES-R score ≥ 33 and “PTSD -” with a low probability of PTSD corresponding to subjects with a total IES-R score < 33. Psychological help was systematically offered to PTSD + patients at the end of the study.

The secondary endpoint was the quality of life assessment through mental and physical quality of life scores, based on the Short Form 12 (SF-12) scale. SF-12 is a 12-items questionnaire, commonly used to calculate a Physical Component Summary (PCS), and a Mental Component Summary (MCS).

Pre-defined potential risk factors were collected through prospectively collected data from the registry and telephone interviews: 1) Demographic characteristics: age, gender, professional activity related to mountain activities and isolation (defined by single person without child); 2) Factors related to the patient’s clinical history: pre-accident psychiatric condition, past situation of avalanche, mountain accident or significant trauma; 3) Factors related to the avalanche episode: severity of trauma assessed using the Injury Severity Score (ISS); pain management by rescue team (use of Morphine or Ketamine); out-of-hospital or in-hospital cardiac arrest; loss of consciousness; transfer to an Intensive Care Unit (ICU); the use of benzodiazepines during hospitalization or within one month after the accident; time between avalanche accident and phone interview; type of activity practiced when the avalanche occurred; burial type: “complete burial” (head under the snow) or “partial burial” (head out of the snow); burial depth; and presence of a death directly caused by the avalanche.

Subjects’ Outcomes were assessed after hospitalisation: Glasgow Outcome Scale [20]; consultation with a general practitioner or a psychologist; antidepressant treatment; time between the avalanche accident and the restart of mountain sport activities.

Finally, we also wondered if avalanche safety equipment was carried during the accident and if new safety equipment was purchased after the avalanche incident.

### Statistical analysis

The prevalence of post-traumatic stress disorder is presented in percent with 95% Confidence Interval (CI). Quantitative data are presented as median and interquartile range (IQR). Qualitative data are presented as frequency and percentage. Proportions were compared using Chi2 test or Fischer’s exact test as appropriate. Means were compared using the Student t-test, or the Mann-Whitney test in case of deviation from the normal distribution. The association between two quantitative data was assessed using the Pearson correlation test. PTSD risk factors were investigated regarding the total score at the IES-R as a continuous variable and after transforming the IES-R scale score into a binary qualitative variable (PTSD + versus PTSD -). Independent risk factor were searched with a multivariate logistic regression including patients and avalanche characteristics as well as clinical management variables with a *p*-value ≤0.10 in univariate analysis. All tests were bilateral with an alpha risk set to 0,05. All statistical analyses were performed using IBM SPSS v.25 software (IBM statistics, USA).

## Results

**Fig. 1:**
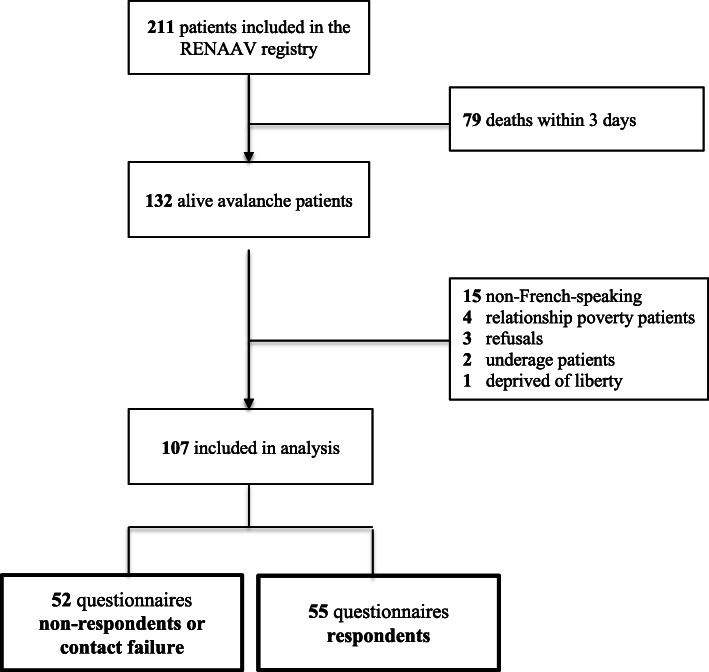
Study Flow chart

Between December 2014 and May 2018, among the 211 avalanche victims included in the RENAAV register, 79 victims died. One hundred and seven victims were successfully contacted, and 55 agreed to participate to phone interviews (Fig. 1). No additional deaths occurred among survivors during the study period. There was no significant difference between respondents and non-respondents subjects regarding population characteristics, medical management and avalanche characteristics (Table 1). Among included victims, 49 (89.1%) were practicing in mountain sports as hobbies, and 6 (10.9%) as professional (military or mountain guide). The avalanche occurred during off-piste or ski touring in 48 (87.3%) cases. None of the victims included in the study underwent a cardiac arrest during the avalanche accident. Among the 55 questionnaire respondents, 48 (87.3%) restarted the same activity, with a median time to return to mountain sport of 7.5 months (Interquartile range -IQR- 0.5 – 10.1). In 60.0% of cases, the victims were equipped with an avalanche transceiver, shovel and probe, and in 20.0% with an airbag backpack. Ten (18.1%) had no safety or rescue equipment during the avalanche. Among these 55 victims, 10 (18.2%) purchased safety or rescue equipment after their avalanche incident.

**Table 1:** Characteristics of the study population

	Included ***n*** = 55	Non included ***n*** = 52	Total ***n*** = 107	p-value*
**Demographic characteristics**				
**Age,** mean ± SD	36.3 ± 11.6	35.9 ± 12.9	36.1 ± 12.2	0.87
**Men,** n (%)	45 (81.8)	48 (92.3)	93 (86.9)	0.10
**Medical characteristics and management**				
**ISS Score,** mean ± SD	9.84 ± 12.76	7.94 ± 13.72	8.92 ± 13.21	0.46
**Intubation,** n (%)	4 (7.3)	1 (1.9)	5 (4.5)	0.36
**ICU admission,** n (%)	9 (16.4)	3 (5.9)	12 (11.3)	0.08
**Avalanche characteristics**				
**Multiple involved victims,** n (%)	31 (56.4)	37 (71.2)	68 (63.6)	0.11
**Concomitant Death in the avalanche,** n (%)	6 (10.9)	12 (23.1)	18 (16.8)	0.09
**Burial type**				
*Partial,* n (%)	36 (65.5)	37 (71.2)	73 (68.2)	
*Complete,* n (%)	19 (34.5)	15 (28.8)	34 (31.8)	0.52
Depth**, mean in meter ± SD	0.89 ± 0.62	0.92 ± 0.61	0.90 ± 0.60	0.91
Time**, mean in minute ± SD	11.53 ± 8.79	6.43 ± 4.45	9.07 ± 7.39	0.06

*Chi-2 or Fisher's exact test when appropriate, Student t-test for means. ** Depth and time of burial were reported only for victims who were completely buried. *SD* Standard Deviation; *ISS* Injury Severity Score; *ICU* Intensive Care Unit

Of the 55 questionnaire respondents, 23 (41.8%) patients consulted their general practitioner after the avalanche incident, 18 (32.7%) had at least one consultation with a psychological specialist, and 3 (5.5%) received antidepressant treatment after the episode. No psychological discomfort during the phone interview was reported.

### IES-R (post-traumatic stress evaluation)

Median total IES-R score was 15 (IQR 9–26). Six patients presented a total IES-R score ≥ 33, indicating a very high probability of PTSD and one additional patient had received specialized care to confirmed PTSD. Therefore, the observed prevalence of PTSD was 10.9% (95% CI 1.76–20.05). Thirteen patients (25%) reported a feeling of imminent death during the accident. Delays between accident and interview for PTSD+ victims were respectively 4 years, 3 years, 2 years (for 2 patients) and 1 year (for 2 patients).

### SF-12 (quality of life evaluation)

The median MCS score was 40.1 (IQR 36.5–43.4) in PTSD- victims, and 39.0 (IQR 30.5–46.3) in PTSD+ victims, *p* = 0.76. The median PCS score was 44.2 (IQR 39.1–46.8) and 39.4 (IQR 37.2–44.3) in PTSD- and PTSD+ victims respectively (*p* = 0.39).

### Risks factors

**Fig. 2:**
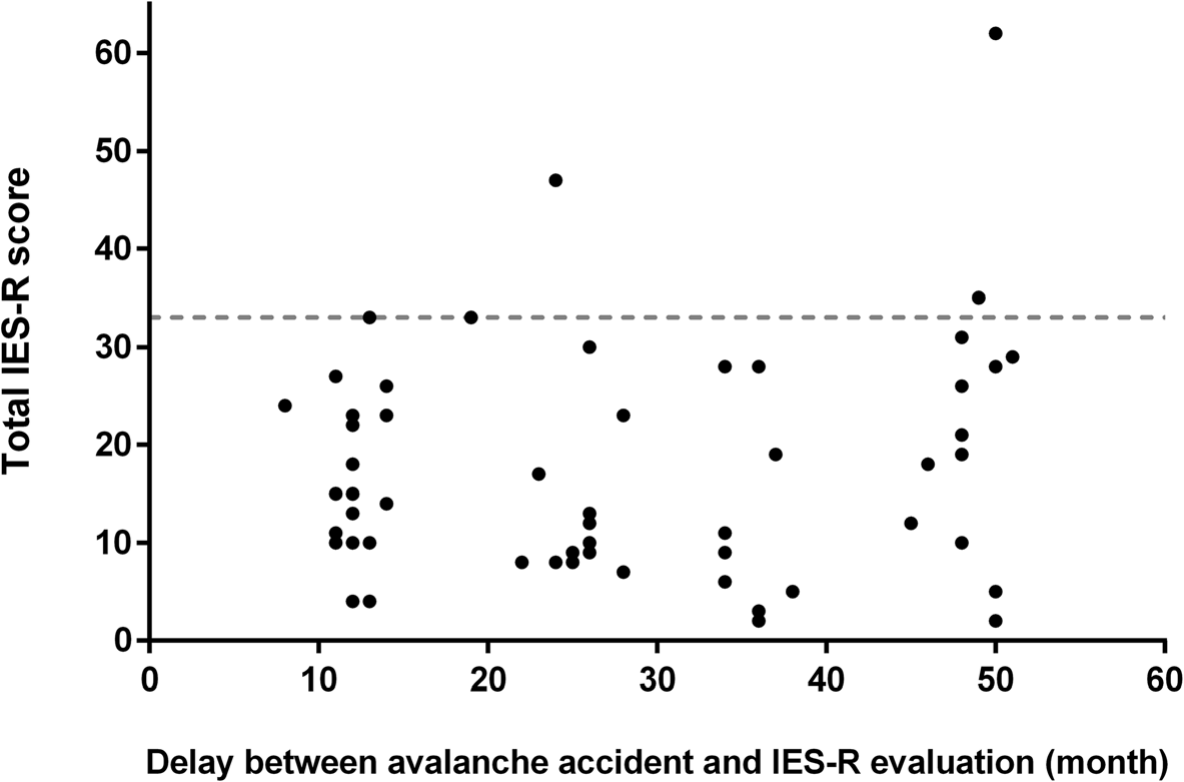
IES-R score according to the delay between avalanche accident and interview. The grey dashed line represents IES-R cut-off score of 33, corresponding to high probability of PTSD

In univariate analysis (Table 2), the complete burial (83.3% in PTSD+ vs 28.6% in PTSD-, *p* = 0.02) was associated with severe PSTD symptoms (IES-R ≥ 33). The total IES-R score was significantly higher for victims who had had another mountain accident (21.43 ± 11.08 vs 15.09 ± 11.62, *p* = 0.02), and for victims who were intubated (36.75 ± 22.62 vs 16.00 ± 9.28, *p* = 0.04) (Table 3). No relationship was observed between the total IES-R score and the delay between the accident and the interview (Fig. 2) or with the Injury Severity Score. In multivariate analysis, no studied factor emerged as an independent risk factor.

**Table 2:** Characteristics of avalanche victims with or without posttraumatic stress disorder according the IES-R score

	PTSD + ***n*** = 6	PTSD - ***n*** = 49	*p*-value*
**Patient characteristics**			
Age, mean ± SD	36.8 ± 13.8	36.2 ± 11.5	0.59
Men, n (%)	4 (66.7)	41 (83.7)	0.30
Profession related to the mountains, n (%)	2 (33.3)	19 (38.8)	0.99
Family situation, n (%)			
*Surrounded*	4 (66.7)	33 (67.3)	0.99
*Isolated*	2 (33.3)	16 (32.7)	
Psychiatric history, n (%)	0 (0)	5 (10.2)	0.99
Avalanche history, n (%)	1 (16.7)	7 (14.3)	0.99
Other mountain accident, n (%)	4 (66.7)	17 (34.7)	0.19
Other trauma, n (%)	2 (33.3)	12 (24.5)	0.64
**Avalanche characteristics**			
Time since the avalanche, mean in month ± SD	31.8 ± 15.6	27.3 ± 14.3	0.41
Multiples involved victims, n (%)	4 (66.7)	27 (55.1)	0.69
Death in the avalanche, n (%)	0 (0)	6 (12.2)	0.99
Burial, n (%)			
***Complete***	**5 (83.3)**	**14 (28.6)**	**0.02**
Depth**, mean in meter ± SD	0.77 ± 0.25	0.93 ± 0.69	0.73
Time**, mean in minute ± SD	7.33 ± 2.52	12.58 ± 9.55	0.45
Activity during the avalanche, n (%)			
*Alpine skiing*	2 (33.3)	1 (2.0)	0.03
*Alpine off-piste skiing*	2 (33.3)	20 (40.8)	0.99
*Touring skiing*	2 (33.3)	25 (51.0)	0.67
*Alpinism*	0 (0.0)	1 (2.0)	0.99
*Hiking (with or without snowshoe)*	0 (0.0)	1 (2.0)	0.99
*Unknown*	0 (0.0)	1 (2.0)	0.99
**Clinical consequences and medical management**			
Trauma lesion, n (%)	5 (83.3)	37 (75.5)	0.99
ISS, n (%) *≥ 15*	1 (16.7)	10 (20.4)	0.99
Loss of consciousness, n (%)	2 (33.3)	9 (18.4)	0.59
Pain management, n (%)			
*Opioids*	1 (16.7)	15 (31.9)	0.65
*Ketamine*	1 (16.7)	8 (17.0)	0.99
Intubation, n (%)	2 (33.3)	2 (4.1)	0.06
ICU, n (%)	2 (33.3)	7 (14.3)	0.25
Benzodiazepines, n (%)	1 (16.7)	3 (6.1)	0.37
**Patient’s outcomes**			
Exit***, n (%)			
*Home*	3 (75.0)	44 (91.7)	0.34
*Rehabilitation*	1 (25.0)	4 (8.3)	
Incomplete recovery, n (%)	1 (16.7)	15 (30.6)	0.66

*Chi-2 or Fisher’s exact test when appropriate, Student t-test for means. **Depth and time of burial were reported only for victims who were completely buried. ***Not admitted or discharge after hospital stay. *IES-R* Impact of Event Scale Revised; *PTSD* posttraumatic stress disorder; *SD* Standard Deviation; *ISS* Injury Severity Score; *ICU* Intensive Care Unit

**Table 3:** Total IES-R score according risks factors

	Total IES-R score	***p-value***
**Patient characteristics**		
Sex		
*Men*	20.30 ± 9.72	
*Women*	16.89 ± 12.14	0.19
Profession related to the mountains		
*Yes*	19.57 ± 13.28	
*No*	16.24 ± 10.68	0.39
Family situation		
*Surrounded*	17.81 ± 13.03	
*Isolated*	16.89 ± 8.80	0.84
Psychiatric history		
*Yes*	11.80 ± 9.18	
*No*	18.08 ± 11.88	0.18
Avalanche history		
*Yes*	20.25 ± 13.83	
*No*	17.04 ± 11.44	0.55
Other mountain accident		
*Yes*	**21.43 ± 11.08**	
*No*	**15.09 ± 11.62**	**0.02**
Other trauma		
*Yes*	17.64 ± 13.57	
*No*	17.46 ± 11.23	0.82
**Avalanche characteristics**		
Number of people involved		
*One*	15.08 ± 8.80	
*Several*	19.39 ± 13.42	0.27
Deadly avalanche		
*Yes*	17.83 ± 10.15	
*No*	17.47 ± 12.01	0.78
Burial		
*Partial*	15.11 ± 8.34	
*Complete*	22.05 ± 15.63	0.11
**Clinical consequences and medical management**		
Traumatic lesion		
*Yes*	16.24 ± 10.19	
*No*	21.62 ± 15.52	0,25
ISS		
*< 15*	17.16 ± 11.96	
*≥ 15*	18.91 ± 11.18	0.60
Loss of consciousness		
*Yes*	20.64 ± 16.29	
*No*	16.73 ± 10.40	0.52
Pain management		
*Opioids*	16.06 ± 11.48	0.46
*Ketamine*	17.11 ± 13.84	0.61
Intubation		
*Yes*	**36.75 ± 22.62**	
*No*	**16.00 ± 9.28**	**0.04**
ICU		
*Yes*	23.89 ± 18.72	
*No*	16.26 ± 9.65	0.35
Benzodiazepines		
*Yes*	**34.75 ± 18.28**	
*No*	**16.16 ± 10.15**	**0.01**
**Patient’s outcomes**		
Discharge		
*Home*^a^	15.91 ± 11.17	
*Rehabilitation*	25.00 ± 14.02	0.12
Glasgow Outcome *Scale*		
*Complete recovery*	17.82 ± 11.81	
*Incomplete recovery*	16.75 ± 11.90	0.65

Values are expressed in mean ± SD. ^a^Not admitted or discharge at home after hospital stay

*IES-R* Impact of Event Scale Revised; *ISS* Injury Severity Score; *ICU* Intensive Care Unit

## Discussion

To the best of our knowledge, this is the first quantitative study focusing on psychological consequences of avalanches among people involved during mountain recreational activities, and based on an exhaustive registry. Previous studies mainly focused on soldiers [13, 16] or on avalanches that buried habitations [11, 14, 21]. Our study was based on the Northern French-Alps Avalanche Registry, which is an exhaustive database where any kinds of avalanche victims are included. In this study population, 10.1% of avalanche survivors presented high probability of PTSD. Additionally, a quarter of patients reported a feeling of imminent death in their accident, which is known to be the main trigger for post-traumatic stress disorder [1]. Avalanche appears to be a brutal event that may cause post-traumatic stress in survivors. This prevalence is similar to the available literature about non-recreational avalanche accidents [13, 15]. In a recent review, Greene et al. showed that 2 to 15% of individuals in the general population suffered from PTSD, regardless of the trauma [5]. In a meta-analysis, Ophuis et al. identified post-traumatic stress one year after any physical trauma in 16 to 27% of victims [22]. Qureshi et al. found PTSD in 21% of patients 18 months after a head injury [23]. Finally, two meta-analyses found a prevalence of post-traumatic stress ranging from 3.7 to 60%, two years after a natural disaster [3, 4]. This wide distribution of prevalence is explained by the multiplicity of measurement tools used and by the variability in the time interval between trauma and PTSD assessment.

In our study, complete burial and intubation tend to be associated with higher IES score. Patients completely buried without air pocket frequently described a feeling of asphyxia. Complete burial and long burial time are already identified as mortality factors [24]. We observed a higher total score at the IES-R in intubated patients. Several studies have shown that mechanical ventilation and administration of narcotics or benzodiazepines were associated with the development of PTSD symptoms [25–27]. On the contrary, in our data, admission to an Intensive Care Unit was not significantly associated with the development of PTSD, although several authors report them as risk factors [28–31]. Unfortunately we failed to identify independent risk factors in this cohort.

Concerning analysis of sports practices, our results are consistent with national data [32]. Most patients were skiing out of slopes in non-secured areas, exposing themselves to more accidents. During interviews, 80% of patients declared being equipped with appropriate safety and rescue equipment indicating a good risk assessment.

We used the IES-R score, efficient to predict PTSD with a cut-off equal or greater than 33 [33], and commonly used for PTSD screening [34]. However, the IES-R does not explore the impact of trauma on mood and cognitive functions. Indeed, many patients reported guiltiness (towards themselves and/or for others). Some patients reported a feeling of isolation and misunderstanding by their relatives. Those factors are recognized as risk factors for PTSD [5, 35, 36]. Three patients also reported memory problems following the avalanche. Concerning victim’s quality of life, we did not manage to highlight a difference unlike other studies. Median quality of life scores were not significantly lower for patients with a high probability of PTSD as compared to patients without PTSD. This is likely due to the lack of power and the relatively low proportion of responders. However, median scores were comparable to the literature data, confirming the impact of an avalanche accident on the victims’ quality of life. In 2017, Falkenberg et al. compared two cohorts of multiple trauma patients according to the presence of PTSD symptoms detected with IES-R [37]. Ages of the two cohorts were comparable to our population. Those with PTSD had an average MCS score of 47.5 and a PCS score of 39.8. Patients without PTSD had significantly higher MCS and PCS scores, 53.6 and 46.4, respectively. In 2011, Westphal et al. assessed quality of life in three groups of patients: those with PTSD in treatment, those with PTSD treated and considered cured, and those with resistant PTSD [38]. The results showed a better quality of life in cured patients (MCS at 42.1 and PCS at 38.7) compared to those with PTSD in treatment (MCS at 34.2 and PCS at 38.0). The primary objective of using SF-12 was to screen patients with significant alterations in their quality of life. To further enhance the impact of the accident, this assessment should be completed with more precise tools.

### Impacts for future practices

The main goal of this study was to raise awareness among medical teams and general practitioners who will have to take care of avalanche survivors. Few strong elements of response exist in the literature concerning the correct attitude or acute treatment to significantly reduce the risk of developing PTSD. The “Debriefing” does not seem to show any beneficial effect and the study could even suggest a negative effect on healing [8]. “Cognitive Behavior Therapy” seems to be the most effective (but with an application delay and an unknown duration), as suggested in the systematic review of Eva Visser et al. [39]. Some authors have proposed to adopt an attitude of “defusing” instead, the effect of which remains to be specified [40–42].. Regarding potential drug treatments, no studies provide a high level of evidence or guidance. Hydrocortisone, Propranolol, Escitalopram, Temazepam, Gabapentine, or opioids have been studied, without clear direction [43].

Nevertheless, early PTSD symptoms should be detected in order to prevent an authentic state of PTSD and its consequences, using a systematic follow-up. We propose a first telephone call one week after the accident by a mountain rescuer for a first diffusing process. Then, two subsequent reminders one month and six months after the avalanche accident by a qualified health care professional could detect PTSD symptoms and to refer patients to an appropriate medical team. Lastly, we propose to screen avalanche rescuers, highly exposed, for post-traumatic stress symptoms [12, 13, 44].

### Limits

Our protocol could have led to an over or underestimation of the prevalence of PTSD. Patients non included could be more affected, or on the contrary could have felt less concerned by the event. First, to ensure the reproducibility of the questionnaires, and not to risk an evaluation biased by a language barrier or slightly different questionnaires, we have also chosen to exclude the 15 living victims out of 132 (11.4%) who could not answer the assessment in French. We cannot guarantee that this does not induce bias, but we believe that this is unlikely given the non-different characteristics of this population. In the same way, half of eligible patients never responded to solicitations, which is common when it comes to actively participating in an assessment with multiple questions. Although we don’t think this could have created a real bias regarding the few or no differences between respondents and non-respondents, we cannot exclude that the patients little affected by the event were less motivated to answer questions about it. This will therefore imply an overestimation of the rate of patients presenting signs of PTSD. Overall, these potential biases are minimized by the absence of clear difference observed in the characteristics of the subjects included or not included in the analysis (Table 1).

Furthermore, time between accident and interview were different. Some patients were interviewed more than 4 years after their accident, inducing a time effect and memory bias. Some patients may have experienced PTSD symptoms more intensely during the months following the accident than several years later. But the lack of correlation between the total IES-R score and the time since the avalanche occurred indicates that PTSD might occur several years after the accident. It would have been better to perform interviews with a standardised delay in a 100% prospective approach.

## Conclusion

Avalanche incidents may induce posttraumatic stress disorders among survivors and affect their quality of life. Mountain Rescue teams should be aware of this risk. Early diagnosis and preventive measures should be set up in order to reduce prevalence of psychological trauma.

## Data Availability

The data that supports the findings of this study are available from the RENAU but restrictions apply to the availability of these data, which were used under license for the current study, and so are not publicly available. Data are however available from the author upon reasonable request and with permission of the RENAU.

## Abbreviations

CI: Confidence Interval
DSM-V: the 5th Edition of the Diagnostic and Statistical Manual of mental disorders
ICU: Intensive Care Unit
IES-R: Impact of Event Scale Revised
IQR: Interquartile Range
ISS: Injury Severity Score
MCS: Mental Component Scale
PCS: Physical Component Scale
PTSD: Post-traumatic Stress Disorder
RENAAV: the Northern French Alps Avalanche Registry
RENAU: the Northern French Alps Emergency Network
SF-12: Short Form 12
